# Cognitive outcome and gamma noise power unrelated to neuregulin 1 and 3 variation in schizophrenia

**DOI:** 10.1186/1744-859X-13-18

**Published:** 2014-06-14

**Authors:** Álvaro Díez, Clara Cieza-Borrella, Vanessa Suazo, Rogelio González-Sarmiento, Sergi Papiol, Vicente Molina

**Affiliations:** 1 Division of Psychiatry, Faculty of Brain Sciences, University College London, London W1W 7EJ, UK; 2 Institute of Biomedical Research of Salamanca (IBSAL), Salamanca 37007, Spain; 3 Molecular Medicine Unit, Department of Medicine and Institute of Molecular and Cellular Cancer Biology (IBMCC), University of Salamanca & CSIC, Salamanca 37007, Spain; 4 Neuroscience Institute of Castilla y León (INCYL), University of Salamanca, Salamanca 37007, Spain; 5 Anthropology Unit, Animal Biology Department, University of Barcelona, Barcelona 08028, Spain; 6 Clinical Neuroscience Section, Max Planck Institute of Experimental Medicine, Göttingen 37075, Germany; 7 Psychiatry Department, School of Medicine, University of Valladolid, Valladolid 47005, Spain; 8 Psychiatry Service, University Hospital of Valladolid, Avenida Ramón y Cajal, 7, Valladolid 47005, Spain

**Keywords:** Schizophrenia, Brain wave, Auditory evoked potential, NRG1 protein, NRG3 protein

## Abstract

**Background:**

Neuregulins are a family of signalling proteins that orchestrate a broad range of cellular responses. Four genes encoding Neuregulins 1–4 have been identified so far in vertebrates. Among them, Neuregulin 1 and Neuregulin 3 have been reported to contribute to an increased risk for developing schizophrenia. We hypothesized that three specific variants of these genes (rs6994992 and rs3924999 for Neuregulin 1 and rs10748842 for Neuregulin 3) that have been related to this illness may modify information processing capacity in the cortex, which would be reflected in electrophysiological parameters (P3b amplitude or gamma noise power) and/or cognitive performance.

**Methods:**

We obtained DNA from 31 patients with schizophrenia and 23 healthy controls and analyzed NRG1 rs6994992, NRG1 rs3924999 and NRG3 rs10748842 promoter polymorphisms by allelic discrimination with real-time polymerase chain reaction (PCR). We compared cognitive outcome, P300 amplitude parameters and an electroencephalographic measure of noise power in the gamma band between the groups dichotomized according to genotype.

**Results:**

Contrary to our hypothesis, we could not detect any significant influence of variation in Neuregulin 1/Neuregulin 3 polymorphisms on cognitive performance or electrophysiological parameters of patients with schizophrenia.

**Conclusions:**

Despite our findings, we cannot discard that other genetic variants and, more likely, interactions between those variants and with genetic variation related to different pathways may still influence cerebral processing in schizophrenia.

## Background

It is possible that the link between genetic variation in neuregulin (NRG) and the increased risk of developing schizophrenia could be established through the influence NRG has on the regulation of distributed cerebral activity. NRGs are a family of signalling proteins containing an epidermal growth factor (EGF)-like domain that activates ErbB receptor-tyrosine kinases, which orchestrate a broad range of cellular responses [1-3]. Among them, Neuregulin 1 (NRG1) has a significant impact upon differentiation, migration and maturation of gamma-Aminobutyric acid (GABA) interneurons [4]. The influence attributed to NRG1-ErbB4 signaling network may have relevant consequences on information processing in the brain, as suggested by the enhancing effect NRG1 displayed on gamma oscillations [5].

Genetic variation in NRG1 was associated to an increased risk for developing schizophrenia [6]. Among the several markers in this gene, the single-nucleotide polymorphism (SNP) rs6994992 (SNP8NRG243177) in the promoter region is particularly interesting because of its effect on the expression levels of type-IV NRG1 in human post-mortem brain samples and in luciferase *in vitro* assays [7,8]. Other genetic variants related to NRG1 have been associated to schizophrenia, such as the non-synonymous (Arg/Gln) SNP rs3924999 [9]. This SNP, which maps to exon E344/E178 (Ig domain), has been associated to higher latency of the P300 potential [10].

Less information is available on the biological functions and possible relevance of Neuregulin 3 (NRG3) for mental illness. However, it is well established that NRG3 is a specific ligand for ErbB4 [11] and plays an outstanding role in the development of the cerebral cortex [12]. Four main classes of NRG3 are generated by alternative splicing [13]. Genetic variation in NRG3 (1.1 Mb, chr.10q23.1), rs10748842 at intron 1, has also been reported to increase the risk for schizophrenia and to exert a functional effect on the expression of class II and III NRG3 isoforms [13].

Among the possible electrophysiological correlates for genetic variation in NRG, we can highlight the P300 potential amplitude and cortical noise power. Cortical noise power refers to the amount of scalp-recorded power not temporally locked to stimuli, quantified as the difference in each band between the mean power of single trials and the power magnitude in the averaged potential [14,15]. P3b amplitude decrease and gamma noise power excess have been previously reported in schizophrenia, the latter inversely correlated to cognitive performance and directly to negative symptoms [16-18].

We hypothesized that NRG variants (i.e., rs6994992 and rs3924999 for NRG1 and rs10748842 for NRG3) may modify the information processing capacity in the cortex, which would be reflected in electrophysiological parameters (P3b amplitude or gamma noise power) and/or cognitive performance.

## Methods

We included 31 patients with paranoid schizophrenia (DSM-IV-TR criteria) and 23 healthy controls (HC). This sample partly overlaps with that of our previous reports on noise power in schizophrenia [16,17].

The schizophrenia cases included 10 stable, treated in the long-term, and 21 untreated cases who received a minimal treatment prior to the electroencephalographic (EEG) examination (minimally treated patients; 12 males), of which 12 were first episodes (7 males). During the preceding year, the stable patients had been treated with risperidone (7 cases (2 to 6 mg/day)) or olanzapine (3 cases (5 to 15 mg/day)). Doses and drugs were unchanged during the 3 months preceding EEG recordings. Prior to their inclusion, minimally treated patients had not received any previous treatment (first episode patients) or they had dropped their medications for a period longer than 1 month. Owing to an acute psychotic state of these patients prior to inclusion, we administered a small amount of haloperidol (2 to 4 mg) 2 days before the EEG study, with a wash-out period of approximately 24 h before EEG. The objective was to minimize the likely bias of only including patients able to cooperate with the EEG recording during an acute psychotic episode. As we reported elsewhere [16,17], this treatment had no significant effect on gamma noise power measurements.

We scored the clinical status of the patients using the Positive and Negative Syndrome Scale (PANSS) [19]. Marital status was stratified into single (single, divorced, separated) or living in couple, and employment status as employed (currently studying or working) or unemployed (looking for a job or retired for health reasons). Education was quantified as completed academic courses; the number of years within the education system since the first official academic year (usually at 6 years old) until the last completed course before dropping out of the system.

We recruited HC through newspaper advertisements and remunerated their cooperation. They were previously assessed using a semi-structured psychiatric interview by one investigator (VM) to discard major psychiatric antecedents (personal or familial) and treatments.

The exclusion criteria included intelligence quotient (IQ) below 70; a history of any neurological illness; cranial trauma with loss of consciousness; past or present substance abuse, except nicotine or caffeine; the presence of any other psychiatric process or treatment and treatment with drugs known to act on the central nervous system. Toxic use was discarded in patients and HC with the information gathered in the interview and a urinalysis.

We obtained written informed consent from the patients and HC after providing full written information. The research board (University Hospital of Salamanca) endorsed the study according to The Code of Ethics of the World Medical Association (Declaration of Helsinki).

### Cognitive assessment

We acquired cognitive assessment using the direct scores from the following subscales of the Spanish version of Brief Assessment in Cognition in Schizophrenia Scale (BACS) [20], administered by trained researchers (VS, ÁD): verbal memory (words list learning), working memory (digit span), motor speed (token motor task), verbal fluency (categories), attention and processing speed (symbol coding), and executive function/problem solving (tower of London). We used the Spanish version of the Wechsler Adult Intelligence Scale 3rd Edition (WAIS-III) [21] to assess IQ.

## EEG methods

The EEG methods have been reported in detail elsewhere [16,17]. Essentially, EEG recordings were performed during an odd-ball task in which P3a and P3b were elicited and measured in the Pz site (see Additional file 1 for details). The EEG was recorded by the Brain Vision® (Brain Products GmbH; Munich, Germany) equipment from 17 tin electrodes mounted in an electrode cap (Electro-Cap International, Inc.; Eaton, OH, USA), impedance kept under 5 kΩ. The on-line register was referenced over Cz electrode and re-referenced off-line to the electrodes' average activity [22]. The sampling rate was 250 Hz, and the signal was recorded continuously. We selected noise power values at F3, F4, P3 and P4 electrodes for the analysis according to our *a priori* hypothesis. See Additional file 1 for detailed information on EEG recording.

For quantitative event-related EEG analysis, the recorded signals (−50 to 600 ms post-stimulus, target condition) were submitted to specific band filtering and spectrum analysis using a fast Fourier transform yielding spectral values. The absolute magnitude (averaged total power) in gamma band (35 to 45 Hz) was computed and expressed in μV^2^.

As described in previous articles [16,17], we calculated noise magnitude, which is subsequently denoted as ‘noise power’. This calculation was based on signal-to-noise ratio (SNR), a measure of the quality of the EEG signal applied to each band; it is calculated using the Brain Vision® software (Brain Products GmbH; Munich, Germany) [23] for the time window from −50 to 600 ms for the target stimuli.

For every individual participant, band and electrode, we calculated the averaged noise power from the already extracted average total power (the sum of the signal and noise power) and SNR (the average signal power quotient divided by the average noise power) using the following formula:

$$\mathrm{Avg}\;\mathrm{noise}\;\mathrm{power}=\frac{\mathrm{Avg}\;\mathrm{total}\;\mathrm{power}}{\mathrm{SNR}+1}$$

This way, a quantification of the noise part of the activity related to the event is approximated and ‘noise’ is equivalent with activity that is not time-locked to the stimuli (see Additional file 1 for details).

### Genetic methods

#### DNA extraction

High molecular weight DNA obtained from peripheral leukocyte blood was isolated using standard procedures with proteinase K and phenol-chloroform purification.

We extracted buccal cells using simple and sterile swabs, without medium (Aptaca, Canelli, Italy). The procedure for the DNA extraction was similar to the protocol for obtaining DNA from peripheral blood except for the proteinase K incubation, which in this case was longer.

Some of the samples extracted from buccal cells have shown a very low DNA concentration. In these cases, we amplified genomic DNA with DNA IllustraTM Amplication GenomiPhi V2 Kit (GE Healthcare, Buckinghamshire, UK).

#### Allelic discrimination by real-time PCR

The analysis of NRG1 rs6994992, NRG1 rs3924999 and NRG3 rs10748842 promoter polymorphisms was carried out using allelic discrimination with real-time polymerase chain reaction (PCR). The final volume of the reaction consisted of 10 μl and we used commercial mixtures of primers and TaqMan MGB® probes (C_22019_10, C_359159_10 and C_1266043_10, respectively) with the sequences VIC: AAGCACCATGCAGGGTTCAAGTGAA**C**GTATACTGGAGGCCAGACCTGCCCA and FAM: AAGCACCATGCAGGGTTCAAGTGAA**T**GTATACTGGAGGCCAGACCTGCCCA for the first polymorphism; VIC: TTTCTTCTTTTAGCCTTGCCTCCCC**A**ATTGAAAGAGATGAAAAGCCAGGAA and FAM: TTTCTTCTTTTAGCCTTGCCTCCCC**G**ATTGAAAGAGATGAAAAGCCAGGAA for the second one; and VIC: TTGCATTGTGAGAGGCTCTGAGTAA**C**TTATGATAAATAATGAAATGCTGTT and FAM: TTGCATTGTGAGAGGCTCTGAGTAA**T**TTATGATAAATAATGAAATGCTGTT for the third (Applied Biosystems, Foster City, CA, USA). We used TaqMan® Universal PCR Master Mix No AmpErase® UNG (Applied Biosystems, Foster City, CA, USA) containing the DNA polymerase. The amplification program had a denaturing temperature of 94°C, annealing of 60°C and extension of 72°C for 1 min each during 40 cycles.

### Statistical analyses

By using Chi squared test or Student's *t* test for independent samples when appropriate, we compared demographic scores between groups. We also used Student's *t* test for comparing neurocognitive and neurophysiological parameters between patients and HC groups.

We studied the possible effect of long-term illness and medication over cognition and neurophysiology by comparing chronic and minimally treated patients' outcome using a non-parametrical test (Mann-Whitney's *U* test).

In order to test the study's main hypotheses (differences in cognition, gamma noise power and P300 parameters related to genetic variation), we dichotomized the variable rs6994992 into carriers (*T*+) or non-carriers (*T*−) of this risk allele (i.e., TT and TC group vs. CC group). Variable rs3924999 variation was dichotomized into carriers and non-carriers of the A allele (AA and AG vs. GG homozygous). As for NRG3 rs10748842, our sample only included TT and CT participants.

We used Mann-Whitney's *U* tests to compare total IQ and BACS scores, P300 amplitude (P3a and P3b) and gamma noise power at F3, F4, P3 and P4 electrodes between the groups dichotomized according to their genotype. Moreover, using the same test, we compared each group of patients with the HC carrying the same variation. We repeated these analyses only including the minimally treated patients' subgroup in order to discard the effects of treatment and chronicity on the arising differences. However, on account of our sample size, the results yielded by these secondary analyses were only considered as informational. Given the number of planned comparisons (gamma noise power in four electrodes, P3a and P3b amplitudes and 6 cognitive variables × 3 genetic variants = 36 comparisons), we set the level of corrected significance at *p* = 0.001 (0.05 × 36 = 0.00138). Accordingly, trend level was set at *p* = 0.006. These restrictions were not applied to the initial comparisons between patients and HC that did not consider genotype because these comparisons were descriptive and did not explore the study's hypothesis.

Finally, taking into account our limited sample size, we performed a power analysis for each of the comparisons involving genetic variants (Bonferroni-corrected significance level of *p* = 0.001; see Additional file 1 for details).

## Results

### Patients vs. HC comparisons

There were no significant differences in age (*t* = 1.306, *df* = 52, *p* = 0.197) or sex distribution (*χ*^2^ = 0.013, *df* = 1, *p* = 0.910) between patients and HC. Marital (*χ*^2^ = 7.014, *df* = 1, *p* = 0.008) and employment status (*χ*^2^ = 5.994, *df* = 1, *p* = 0.014) were significantly different between groups. IQ and all BACS scores were significantly lower in patients (Table 1).

**Table 1:** Sociodemographic, clinical, cognitive and neurophysiological values in patients and healthy controls

	**All patients (*N*** **= 31)**	**Healthy controls (*N*** **= 23)**
Age	36.42 (12.07)	31.91 (13.14)
Sex (M:F)	18:13	13:10
Marital status (% singles)**	96.30	68.18
Employment status (% employed)*	42.31	77.27
Education (completed courses)	10.94 (3.81)	11.50 (3.00)
Total IQ**	81.68 (14.20)	104.00 (13.09)
PANSS positive	20.25 (4.33)	N/A
PANSS negative	19.68 (5.54)	N/A
PANSS total	75.46 (12.11)	N/A
BACS - verbal memory***	36.18 (12.38)	54.87 (7.68)
BACS - working memory***	16.93 (5.16)	22.17 (3.77)
BACS - motor speed***	50.24 (15.52)	64.61 (14.85)
BACS - verbal fluency***	16.41 (4.76)	25.48 (4.84)
BACS - processing speed***	37.32 (14.72)	58.57 (12.24)
BACS - problem solving***	12.33 (6.12)	17.52 (3.10)
P3b valid segments (n)	44.87(21.05)	57.13(24.48)
Pz amplitude S2 (P3a; μV)	0.870 (1.125)	1.297 (0.981)
Pz amplitude S3 (P3b; μV)**	0.815 (1.587)	1.935 (0.910)
Gamma noise power F3 (μV^2^)	0.012 (0.012)	0.009 (0.015)
Gamma noise power F4 (μV^2^)	0.012 (0.013)	0.008 (0.009)
Gamma noise power P3 (μV^2^)**	0.012 (0.009)	0.006 (0.004)
Gamma noise power P4 (μV^2^)**	0.012 (0.007)	0.007 (0.004)

Data specified as percentage (%), simple distribution or pertaining to Mean (S.D.); *p < 0.05; **p < 0.01; ***p < 0.001 (Chi square test or Student's *t* test when corresponding); N/A, not applicable.

Patients showed a significant reduction in P3b amplitude (*t* = 3.029; *df* = 52; *p* = 0.004) and a significantly higher gamma noise power at P3 (*t* = 2.971; *df* = 52; *p* = 0.004) and P4 (*t* = 2.669; *df* = 52; *p* = 0.010) electrodes in comparison to HC (Table 1).

### Chronic vs. minimally treated patients

Chronic patients were significantly older and had more negative symptoms than minimally treated patients (*p* < 0.01). There were no significant differences between these groups in the main cognitive and neurophysiological measures (Additional file 1: Table S1).

### Genetic-related differences

#### NRG1 rs6994992

There were 21 *T* + (TT homozygous or TC heterozygous) and 10 *T* − (CC) in the patient group (13 and 8 respectively for minimally treated patients). In the HC group, there were 12 *T* + and 11 *T*−. This difference was not significant (*χ*^2^ = 1.347, *df* = 1, *p* = 0.246).

There were no significant differences in cognitive performance between *T* + vs. *T* − patients or between *T* + and *T* − HC, even at uncorrected *p* < 0.05 level (Table 2). Power values were low in all cases (*P* < 18%) (Additional file 1: Table S3).

**Table 2:** Sociodemographic, clinical, cognitive and neurophysiological values according to NRG1 rs6994992 and rs3924999, and NRG3 rs10748842 genotypes

	**NRG1 rs6994992**				**NRG1 rs3924999**				**NRG3 rs10748842**			
	**Patients**		**Healthy controls**		**Patients**		**Healthy controls**		**Patients**		**Healthy controls**	
	***T*** **+ (*N*** **= 21)**	***T*** **− (*N*** **= 10)**	***T*** **+ (*N*** **= 12)**	***T*** **− (*N*** **= 11)**	***A*** **+ (*N*** **= 18)**	***A*** **− (*N*** **= 13)**	***A*** **+ (*N*** **= 16)**	***A*** **− (*N*** **= 7)**	***C*** **+ (*N*** **= 8)**	***C*** **− (*N*** **= 23)**	***C*** **+ (*N*** **= 4)**	***C*** **− (*N*** **= 19)**
Age (years)	36.24 (12.26)	36.80 (12.33)	33.92 (14.86)	29.73 (11.25)	35.50 (14.02)	37.69 (9.10)*	33.69 (13.42)	27.86 (12.44)	35.38 (8.40)	36.78 (13.26)	40.25 (21.22)	30.16 (10.82)
Total IQ	82.16 (15.85)***	80.67 (10.62)**	101.92 (11.91)	106.27 (14.50)	81.13 (14.74)***	82.42 (14.04)**	104.19 (14.10)	103.57 (11.47)	83.67 (10.58)*	81.14 (15.20)***	106.25 (14.34)	103.53 (13.19)
PANSS positive	20.05 (3.56)	20.75 (6.14)	N/A	N/A	20.94 (4.48)	19.33 (4.14)	N/A	N/A	19.86 (5.11)	20.38 (4.18)	N/A	N/A
PANSS negative	20.10 (5.77)	18.63 (5.13)	N/A	N/A	17.69 (5.10)*	22.33 (5.14)	N/A	N/A	20.29 (7.89)	19.48 (4.75)	N/A	N/A
PANSS total	75.75 (11.00)	74.75 (15.37)	N/A	N/A	71.88 (11.62)	80.25 (11.48)	N/A	N/A	75.00 (11.66)	75.62 (12.53)	N/A	N/A
BACS verbal memory	35.44 (14.14)**	37.50 (8.90)***	51.75 (8.71)	58.27 (4.69)	37.53 (13.30)***	34.09 (11.09)*	55.50 (6.61)	53.43 (10.18)	34.86 (14.04)	36.62 (12.12)***	50.25 (9.91)	55.84 (7.07)
BACS working memory	16.33 (5.82)**	18.11 (3.48)*	22.17 (4.34)	22.18 (3.25)	17.13 (5.15)**	16.67 (5.38)	22.50 (3.18)	21.43 (5.09)	16.83 (4.54)*	16.95 (5.43)**	23.00 (1.41)	22.00 (4.11)
BACS motor speed	48.63 (17.03)*	53.30 (12.37)	65.33 (15.69)	63.82 (14.60)	52.18 (14.63)	47.50 (16.95)**	60.75 (14.23)	73.43 (13.10)	50.43 (13.76)	50.18 (16.34)**	59.50 (23.74)	65.68 (12.98)
BACS verbal fluency	16.32 (5.38)***	16.60 (3.53)***	25.17 (5.37)	25.82 (4.42)	16.12 (5.13)***	16.83 (4.34)*	27.13 (3.38)	21.71 (5.79)	17.43 (3.60)**	16.09 (5.10)***	27.75 (4.35)	25.00 (4.91)
BACS processing speed	38.33 (17.47)**	35.50 (8.25)***	57.17 (11.47)	60.09 (13.42)	38.24 (16.74)***	35.91 (11.53)**	60.13 (13.02)	55.00 (10.21)	37.86 (14.46)	37.14 (15.15)***	49.75 (5.44)	60.42 (12.54)
BACS problem solving	13.41 (5.48)	10.50 (7.00)*	16.92 (3.06)	18.18 (3.16)	11.50 (6.20)**	13.55 (6.09)	17.25 (3.32)	18.14 (2.67)	12.86 (5.61)	12.15 (6.42)**	15.75 (3.77)	17.89 (2.92)
P3b valid segments (n)	45.76 (18.53)	43.00 (26.62)	53.25 (25.03)	61.36 (24.33)	43.00 (21.01)	47.46 (21.68)	54.94 (25.72)	62.14 (22.41)	47.38 (27.51)	44.00 (18.99)	54.00 (17.42)	57.79 (26.07)
Pz amplitude S2 (P3a; μV)	0.968 (1.127)	0.666 (1.154)	1.485 (0.762)	1.093 (1.179)	0.929 (1.193)	0.789 (1.066)	1.210 (0.982)	1.497 (1.025)	0.707 (1.052)	0.927 (1.167)	1.114 (0.691)	1.336 (1.043)
Pz amplitude S3 (P3b; μV)	0.735 (1.509)**	0.983 (1.814)	2.033 (0.680)	1.827 (1.136)	0.769 (1.449)*	0.878 (1.821)*	1.623 (0.873)	2.647 (0.532)	0.623 (1.553)*	0.882 (1.628)*	1.915 (0.400)	1.939 (0.993)
Gamma Noise Power F3 (μV^2^)	0.012 (0.012)	0.011 (0.011)	0.007 (0.003)	0.013 (0.022)	0.014 (0.014)*	0.009 (0.005)	0.007 (0.005)	0.016 (0.026)	0.013 (0.012)*	0.011 (0.012)	0.004 (0.000)	0.011 (0.016)
Gamma Noise Power F4 (μV^2^)	0.009 (0.006)	0.018 (0.021)	0.006 (0.003)	0.011 (0.013)	0.015 (0.017)	0.007 (0.003)	0.007 (0.004)	0.012 (0.016)	0.013 (0.016)	0.011 (0.012)	0.005 (0.001)	0.009 (0.010)
Gamma Noise Power P3 (μV^2^)	0.011 (0.009)*	0.015 (0.010)**	0.006 (0.003)	0.007 (0.004)	0.013 (0.010)**	0.012 (0.008)	0.006 (0.004)	0.006 (0.002)	0.013 (0.009)*	0.012 (0.010)**	0.004 (0.002)	0.007 (0.004)
Gamma Noise Power P4 (μV^2^)	0.012 (0.008)*	0.011 (0.005)	0.007 (0.005)	0.007 (0.004)	0.013 (0.009)**	0.009 (0.003)	0.007 (0.004)	0.009 (0.006)	0.011 (0.006)	0.012 (0.008)*	0.005 (0.004)	0.008 (0.005)

No within-group significant differences were detected between patients or HC subgroups classified according to their genotype. Differences between patients and HC with the same SNP variation are displayed in the Patients column. Data exhibited as Mean (S.D.); *p < 0.05; **p < 0.01; ***p < 0.001; Significant results after Bonferroni correction (*p* < 0.00138) are in italics (Mann-Whitney's *U* test); N/A, not applicable.

*T* + and *T* − patients showed worse cognitive performance than the corresponding *T* + and *T* − HC, reaching the required level of significance (*p* < 0.001; *P* > 96%) for total IQ (*T* + patients vs. *T* + HC), verbal memory (*T* − patients vs. *T* − HC), verbal fluency (*T* + patients vs. *T* + HC and *T* − patients vs. *T* − HC) and processing speed (*T* − patients vs. *T* − HC) (Table 2 and Additional file 1: Table S3); in the minimally treated patients considered alone, the level of significance held up at *p* < 0.001 (Additional file 1: Table S2).

Gamma noise power was not significantly different between *T* + and *T* − patients or between *T* + and *T* − HC, with power values always below 7% (Table 2 and Additional file 1: Table S3).

*T* + and *T* − patients did not show significant differences in P3a, P3b or gamma noise power at any electrode site in comparison to *T* + and *T* − HC, with power value below 26% (Additional file 1: Table S3).

#### NRG1 rs3924999

There were 18 *A* + (AA or AG) and 13 *A* − (GG) in the patient group (12 and 9 respectively for minimally treated patients). In the HC group, there were 16 *A* + and 7 *A*−. This difference was non-significant (*χ*^2^ = 0.749, *df* = 1, *p* = 0.387).

There were no significant differences in cognitive performance between A + and A − patients or between *A* + and *A* − HC, even at uncorrected *p* < 0.05 level (Table 2). Power values were below 25% in all cases (Additional file 1: Table S3).

*A* + patients showed significantly worse cognitive performance than the corresponding *A* + HC (*p* < 0.001; *P* > 83%) for total IQ, verbal memory, verbal fluency and processing speed (Table 2 and Additional file 1: Table S3). These differences also held up for the minimally treated patients considered alone (Additional file 1: Table S2).

There were no significant differences in gamma noise power or in P3b and P3a amplitudes between *A* + and *A* − patients or between *A* + and *A* − HC, with power values always below 16% (Table 2 and Additional file 1: Table S3).

*A* + and *A* − patients did not show significant differences in P3a, P3b or gamma noise power at any electrode site in comparison to *A* + and *A* − HC, with power value below 36% (Additional file 1: Table S3).

#### NRG3 rs10748842

There were 8 *C* + (only CT heterocygotes), and 23 *C* − (TT homocygotes) in the patient group (5 and 16 respectively for minimally treated patients). In the HC group, there were 4 *C* + and 19 *C*−. This difference was not significant (*χ*^2^ = 0.541, *df* = 1, *p* = 0.462).

There were no significant differences in cognitive performance between subgroups of patients depending on their genotype, with power values always below 13% (Table 2 and Additional file 1: Table S3).

*C* − patients showed significantly lower total IQ, verbal memory, verbal fluency and processing speed scores than *C* − HC (*p* < 0.001; *P* > 98%; Table 2 and Additional file 1: Table S3). This result was also found in the comparison between minimally treated patients and HC (Additional file 1: Table S2).

There were no significant differences between *C* + and *C* − patients in gamma noise power or P3b and P3a amplitudes (Table 2). Power values were low (*P* < 8%) in all cases (Additional file 1: Table S3).

*C* + and *C* − patients did not show significant differences in P3a, P3b or gamma noise power at any electrode site in comparison to *C* + and *C* − HC, with power value below 26% (Additional file 1: Table S3).

## Discussion

We examined possible abnormal cerebral information processing in a group of chronic and minimally treated patients with schizophrenia using electrophysiological and cognitive data of interest and the association with genetic variation in three SNPs related to increased risk of suffering this disease.

We could not detect any significant influence of variation in NRG1/NRG3 polymorphisms on the cognitive performance of patients with schizophrenia. This negative finding is coherent with previous data, which did not show an association between cognition and genetic variation in NRG in schizophrenia [24]. The variety of functions subserved by NRG in the brain's development suggests the possibility that other SNP or haplotypes related to NRG1 and/or ERBB4 may be associated to cognitive deficit and/or disorganized cortical activity in schizophrenia [25,26].

Variation in NRG polymorphisms included in our study may have a significant effect on other relevant functional parameters in schizophrenia that were not assessed in our study: the T allele for rs6994992 has been associated to prepulse inhibition deficits [27], fronto-temporal activation and cognitive deficits [28], and white matter integrity [29] in schizophrenia.

We did not find an association between electrophysiological parameters of activation (P3b amplitude or gamma noise power) and any of the explored genetic variation in NRG, in spite of previous suggestive evidence obtained with functional magnetic resonance (fMRI) in relation to rs6994992 variant [28]. In the abovementioned study, the authors compared differences in task-related activation, while our aim was to compare the amount of gamma activity unrelated to task performance. Therefore, it seems possible that rs6994992 variant may have an effect on task-related activation but not on basal activity.

Other NRG variants not included in our study may have an influence on relevant activation patterns in schizophrenia. For example, SNP8NRG221533 (rs35753505) was associated to a longer latency [30] of the P300 potential in this illness. In healthy participants, the number of risk alleles of rs35753505 variant correlated with hyperactivation of the frontal gyrus during a working memory test [31] and other regions during memory encoding [32]. Finally, NRG1 5′ and 3′ SNPs rs4560751 and rs3802160 polymorphisms (likelihood ratio test *p* = 0.00020) interacted with schizophrenia, and contributed to inefficient cortical activation during a working memory task in HC [33]. It could be interesting to investigate if these polymorphisms have an effect on EEG variables such as the ones we assessed in this work.

Epistatic effects may contribute to explain the lack of influence of the polymorphisms selected in our study on complex phenotypic features, such as cortical activation and cognition. Previous findings suggest epistasis between NRG1 (rs10503929; Thr286/289/294Met) and its receptor ERBB4 (rs1026882) as well as a three-way interaction with these two SNPs and AKT1 (rs2494734) [33]. Epistatic effects may be especially important between genetic variations concerning NRG1 and, on the other hand, ErbB4 receptors and/or *N*-methyl-d-aspartic acid (NMDA) transmission [25]. However, one of our study limitations is that we did not assess possible epistatic interactions between genotypes and cognitive and electrophysiological parameters.

Another limitation is the possible effects of chronicity and medication on cortical processing, which may mask the interaction with the studied genetic variations. According to our results, chronicity does not seem to alter patients' cognitive or neurophysiological outcome. However, even though we attempted to control for this possible effect by repeating the analyses only including the minimally treated patients' subgroup, the sample size reduction may limit any further conclusions. Finally, our sample size is limited as evidenced by the power analyses, especially for the neurophysiological comparisons between subgroups according to their genetic variants. Therefore, the absence of differences depending on the genotypes might be due to type II errors; nevertheless, this would be contradictory with the similar direction of the exhibited differences between our patients with any variant for each genotype and the corresponding HC.

## Conclusions

We could not detect any significant influence of variation in the studied Neuregulin 1 and Neuregulin 3 polymorphisms on cognitive performance or on gamma noise power magnitudes in patients with schizophrenia. Variation at these loci may have a significant effect on other relevant functional parameters not assessed in our study.

## Abbreviations

BACS: Brief Assessment in Cognition in Schizophrenia Scale; EEG: electroencephalogram; EGF: epidermal growth factor; fMRI: functional magnetic resonance imaging; GABA: gamma-Aminobutyric acid; HC: healthy controls; IQ: intelligence quotient; NMDA: *N*-methyl-d-aspartic acid; NRG: neuregulin; PANSS: Positive and Negative Syndrome Scale; PCR: polymerase chain reaction; SNP: single-nucleotide polymorphism; SNR: signal-to-noise ratio; WAIS-III: Wechsler Adult Intelligence Scale 3rd Edition.

## Competing interests

The authors declare that they have no competing interests.

## Authors' contributions

VM designed the study. VM, ÁD, RG and SP wrote the protocol. VM, ÁD, CC and VS undertook the statistical analysis and writing of the first draft of the manuscript. All authors contributed to the analysis, interpretation, revision and approval of the manuscript.

## Supplementary Material

Additional file 1 **Additional methods and results.** This file includes a description for additional EEG recording methods and analysis (pre-processing, P3a and P3b calculation, and signal-to-noise ratio), power analysis procedure and results for the main contrasts, and study results for minimally treated patients in comparison to chronic patients and healthy controls. Click here for file

## References

[B1] Buonanno A Fischbach GD Neuregulin and ErbB receptor signaling pathways in the nervous system Curr Opin Neurobiol 2001 11 287 296 10.1016/S0959-4388(00)00210-5 11399426

[B2] Burden S Yarden Y Neuregulins and their receptors: a versatile signaling module in organogenesis and oncogenesis Neuron 1997 18 847 855 10.1016/S0896-6273(00)80324-4 9208852

[B3] Falls DL Neuregulins: functions, forms, and signaling strategies Exp Cell Res 2003 284 14 30 10.1016/S0014-4827(02)00102-7 12648463

[B4] Flames N Long JE Garratt AN Fischer TM Gassmann M Birchmeier C Lai C Rubenstein JL Marin O Short- and long-range attraction of cortical GABAergic interneurons by neuregulin-1 Neuron 2004 44 251 261 10.1016/j.neuron.2004.09.028 15473965

[B5] Fisahn A Neddens J Yan L Buonanno A Neuregulin-1 modulates hippocampal gamma oscillations: implications for schizophrenia Cereb Cortex 2009 19 612 618 10.1093/cercor/bhn107 18632742 PMC2638818

[B6] Stefansson H Sigurdsson E Steinthorsdottir V Bjornsdottir S Sigmundsson T Ghosh S Brynjolfsson J Gunnarsdottir S Ivarsson O Chou TT Hjaltason O Birgisdottir B Jonsson H Gudnadottir VG Gudmundsdottir E Bjornsson A Ingvarsson B Ingason A Sigfusson S Hardardottir H Harvey RP Lai D Zhou M Brunner D Mutel V Gonzalo A Lemke G Sainz J Johannesson G Andresson T Neuregulin 1 and susceptibility to schizophrenia Am J Hum Genet 2002 71 877 892 10.1086/342734 12145742 PMC378543

[B7] Law AJ Lipska BK Weickert CS Hyde TM Straub RE Hashimoto R Harrison PJ Kleinman JE Weinberger DR Neuregulin 1 transcripts are differentially expressed in schizophrenia and regulated by 5′ SNPs associated with the disease P Natl Acad Sci USA 2006 103 6747 6752 10.1073/pnas.0602002103 PMC1458952 16618933

[B8] Tan W Wang Y Gold B Chen J Dean M Harrison PJ Weinberger DR Law AJ Molecular cloning of a brain-specific, developmentally regulated neuregulin 1 (NRG1) isoform and identification of a functional promoter variant associated with schizophrenia J Biol Chem 2007 282 24343 24351 10.1074/jbc.M702953200 17565985

[B9] Yang JZ Si TM Ruan Y Ling YS Han YH Wang XL Zhou M Zhang HY Kong QM Liu C Zhang DR Yu YQ Liu SZ Ju GZ Shu L Ma DL Zhang D Association study of neuregulin 1 gene with schizophrenia Mol Psychiatr 2003 8 706 709 10.1038/sj.mp.4001377 12874607

[B10] Kang C Yang X Xu X Liu H Su P Yang J Association study of neuregulin 1 gene polymorphisms with auditory P300 in schizophrenia Am J Med Genet B 2012 159B 422 428 10.1002/ajmg.b.32045 22467496

[B11] Benzel I Bansal A Browning BL Galwey NW Maycox PR McGinnis R Smart D St Clair D Yates P Purvis I Interactions among genes in the ErbB-Neuregulin signalling network are associated with increased susceptibility to schizophrenia Behav Brain Funct 2007 3 31 10.1186/1744-9081-3-31 17598910 PMC1934910

[B12] Assimacopoulos S Grove EA Ragsdale CW Identification of a Pax6-dependent epidermal growth factor family signaling source at the lateral edge of the embryonic cerebral cortex J Neurosci 2003 23 6399 6403 12878679 10.1523/JNEUROSCI.23-16-06399.2003 PMC6740631

[B13] Kao WT Wang Y Kleinman JE Lipska BK Hyde TM Weinberger DR Law AJ Common genetic variation in Neuregulin 3 (NRG3) influences risk for schizophrenia and impacts NRG3 expression in human brain P Natl Acad Sci USA 2010 107 15619 15624 10.1073/pnas.1005410107 PMC2932571 20713722

[B14] Möcks J Kohler W Gasser T Pham DT Novel approaches to the problem of latency jitter Psychophysiology 1988 25 217 226 10.1111/j.1469-8986.1988.tb00992.x 3399610

[B15] Winterer G Ziller M Dorn H Frick K Mulert C Wuebben Y Herrmann WM Coppola R Schizophrenia: reduced signal-to-noise ratio and impaired phase-locking during information processing Clin Neurophysiol 2000 111 837 849 10.1016/S1388-2457(99)00322-3 10802455

[B16] Diez A Suazo V Casado P Martin-Loeches M Molina V Spatial distribution and cognitive correlates of gamma noise power in schizophrenia Psychol Med 2013 43 1175 1185 10.1017/S0033291712002103 22963867

[B17] Suazo V Diez A Martin C Ballesteros A Casado P Martin-Loeches M Molina V Elevated noise power in gamma band related to negative symptoms and memory deficit in schizophrenia Prog Neuro-Psychoph 2012 38 270 275 10.1016/j.pnpbp.2012.04.010 22549114

[B18] Winterer G Coppola R Goldberg TE Egan MF Jones DW Sanchez CE Weinberger DR Prefrontal broadband noise, working memory, and genetic risk for schizophrenia Am J Psychiat 2004 161 490 500 10.1176/appi.ajp.161.3.490 14992975

[B19] Kay SR Fiszbein A Opler LA The positive and negative syndrome scale (PANSS) for schizophrenia Schizophrenia Bulletin 1987 13 261 276 10.1093/schbul/13.2.261 3616518

[B20] Segarra N Bernardo M Gutiérrez F Justicia A Fernández-Egea E Allas M Salfont G Contreras F Gascón J Soler-Insa PA Menchon JM Junque C Keefe RS Spanish validation of the Brief Assessment in Cognition in Schizophrenia (BACS) in patients with schizophrenia and healthy controls Eur Psychiatry 2011 26 69 79 20435446 10.1016/j.eurpsy.2009.11.001

[B21] Wechsler D Wechsler Adult Intelligence Scale 1997 3 San Antonio, TX, USA: The Psychological Corporation

[B22] Bledowski C Prvulovic D Hoechstetter K Scherg M Wibral M Goebel R Linden DE Localizing P300 generators in visual target and distractor processing: a combined event-related potential and functional magnetic resonance imaging study J Neurosci 2004 24 9353 9360 10.1523/JNEUROSCI.1897-04.2004 15496671 PMC6730097

[B23] Brain Vision Analyzer: User Manual 2006 Brain Products GmbH

[B24] Crowley JJ Keefe RS Perkins DO Stroup TS Lieberman JA Sullivan PF The neuregulin 1 promoter polymorphism rs6994992 is not associated with chronic schizophrenia or neurocognition Am J Med Genet B 2008 147B 1298 1300 10.1002/ajmg.b.30727 PMC3875465 18286587

[B25] Banerjee A Macdonald ML Borgmann-Winter KE Hahn CG Neuregulin 1-erbB4 pathway in schizophrenia: from genes to an interactome Brain Res Bull 2010 83 132 139 10.1016/j.brainresbull.2010.04.011 20433909 PMC5050041

[B26] Chen PL Avramopoulos D Lasseter VK McGrath JA Fallin MD Liang KY Nestadt G Feng N Steel G Cutting AS Wolyniec P Pulver AE Valle D Fine mapping on chromosome 10q22-q23 implicates Neuregulin 3 in schizophrenia Am J Hum Genet 2009 84 21 34 10.1016/j.ajhg.2008.12.005 19118813 PMC2668048

[B27] Roussos P Giakoumaki SG Adamaki E Bitsios P The influence of schizophrenia-related neuregulin-1 polymorphisms on sensorimotor gating in healthy males Biol Psychiat 2011 69 479 486 10.1016/j.biopsych.2010.09.009 21035784

[B28] Hall J Whalley HC Job DE Baig BJ McIntosh AM Evans KL Thomson PA Porteous DJ Cunningham-Owens DG Johnstone EC Lawrie SM A neuregulin 1 variant associated with abnormal cortical function and psychotic symptoms Nat Neurosci 2006 9 1477 1478 10.1038/nn1795 17072305

[B29] McIntosh AM Moorhead TW Job D Lymer GK Munoz Maniega S McKirdy J Sussmann JE Baig BJ Bastin ME Porteous D Evans KL Johnstone EC Lawrie SM Hall J The effects of a neuregulin 1 variant on white matter density and integrity Mol Psychiatr 2008 13 1054 1059 10.1038/sj.mp.4002103 17925794

[B30] Bramon E Dempster E Frangou S Shaikh M Walshe M Filbey FM McDonald C Sham P Collier DA Murray R Neuregulin-1 and the P300 waveform–a preliminary association study using a psychosis endophenotype Schizophr Res 2008 103 178 185 10.1016/j.schres.2008.03.025 18571900

[B31] Krug A Markov V Eggermann T Krach S Zerres K Stocker T Shah NJ Schneider F Nothen MM Treutlein J Rietschel M Kircher T Genetic variation in the schizophrenia-risk gene neuregulin1 correlates with differences in frontal brain activation in a working memory task in healthy individuals Neuroimage 2008 42 1569 1576 10.1016/j.neuroimage.2008.05.058 18606232

[B32] Krug A Markov V Krach S Jansen A Zerres K Eggermann T Stocker T Shah NJ Nothen MM Treutlein J Rietschel M Kircher T The effect of Neuregulin 1 on neural correlates of episodic memory encoding and retrieval Neuroimage 2010 53 985 991 10.1016/j.neuroimage.2009.12.062 20036336

[B33] Nicodemus KK Law AJ Radulescu E Luna A Kolachana B Vakkalanka R Rujescu D Giegling I Straub RE McGee K Gold B Dean M Muglia P Callicott JH Tan HY Weinberger DR Biological validation of increased schizophrenia risk with NRG1, ERBB4, and AKT1 epistasis via functional neuroimaging in healthy controls Arch Gen Psychiat 2010 67 991 1001 10.1001/archgenpsychiatry.2010.117 20921115 PMC4291187

