# Supplementary material for: Cognitive outcome and gamma noise power unrelated to neuregulin 1 and 3 variation in schizophrenia
Source: Ann Gen Psychiatry. 2014 Jun 14;13:18. doi: 10.1186/1744-859X-13-18 (PMC4065086; doi:10.1186/1744-859X-13-18)
Supplement: Additional file 1 — Additional methods and results. This file includes a description for additional EEG recording methods and analysis (pre-processing, P3a and P3b calculation, and signal-to-noise ratio), power analysis procedure and results for the main contrasts, and study results for minimally treated patients in comparison to chronic patients and healthy controls. [file 1744-859X-13-18-S1.docx]

# ADDITIONAL FILE 1

## EEG recording:

Participants heard binaural tone bursts (duration 50 ms, rise and fall time 5 ms and intensity 90 dB) presented via speakers with random stimulus onset asynchrony of 1000 and 1500 ms. Random series of 600 tones consisted of target (500 Hz), distracter (1000 Hz) and standard (2000 Hz) tones with probabilities of 0.20, 0.20 and 0.60, respectively. We asked the participants to press a button whenever they detected the target tones, to close their eyes and avoid eye movements and muscle artifacts. The electrode sites were Fp1, Fp2, F3, Fz, F4, F7, F8, C3, Cz, C4, P3, Pz, P4, T5, T6, O1 and O2 of the revised 10/20 International System.

## EEG data Analysis:

***EEG pre-processing:***

We divided the continuous recording into 650 ms epochs starting 50 ms before stimulus onset. We used an off-line 0.5 to 70 Hz filter. Artifacts were automatically rejected by eliminating epochs that exceeded a range of ±70 µV in any of the channels. Based on a visual inspection we eliminated any epochs that still presented artifacts. Individual data were included in the analysis if 50 or more useful epochs were available. Overall, the mean rate of rejected segments was of 50.5%, in an effort to retain the most artifact-free data. We defined baseline as the available 50 ms prestimulus EEG.

### P3a and P3b calculation:

Generation of ERP Grand Averages across montage and subsequent topographic EEG magnitude analyses were automatically performed using Brain Vision Software.

P3 amplitude corresponded to the mean amplitude in the interval 300 to 400 ms following tones’ onsets at the Pz electrode. The P3a and P3b waveforms were extracted respectively from the distracter-related and target-related Grand Averages.

***Signal-to-Noise Ratio calculation:***

Signal-to-noise ratio (SNR) was calculated using the Brain Vision software package (Brain Products GmbH; Munich, Germany). The SNR provides a measure of the quality of the EEG signal. Since neither the signal nor the noise in the EEG is known exactly, average noise power must be estimated with statistical methods.

In this process it is assumed that noise will be eliminated by averaging. Thus average noise power for each channel is calculated from the total of the squares of the differences between the EEG value and the average value, divided by the number of points minus 1:

$$Avg Noise Power=\frac{\sum_{n=1}^{N} \sum_{k=1}^{K} \left( A_{kn}-Ā \right)^{2}}{K*N-1}$$

Where N is the number of segments, K is the data point number in the segment, A_kn_ is the amplitude of each point, and Ā is the average total amplitude (separately for each channel):

$$Ā=\frac{\sum_{n=1}^{N} \sum_{k=1}^{K} A_{kn}}{K*N}$$

The average total power of a channel is a result of the mean of the squares for all data points of the channel before averaging:

$$Avg Total Power=\frac{\sum_{n=1}^{N} \sum_{k=1}^{K} A_{kn}^{2}}{K*N}$$

It can be assumed that the signal and noise are uncorrelated. Consequently the average power of the signal is equal to the difference between the average total power and the average noise power:

$$Avg Signal Power=Avg Total Power-Avg Noise Power$$

The SNR is then calculated from the quotient of the average signal power divided by average noise power.

$$SNR=\frac{Avg Signal Power}{Avg Noise Power}$$

**Power analysis:**

We performed a power analysis (P) using the equation for contrasts between two means (unpaired data):

$$P=Normsdist\left( \left( \sqrt{\frac{nd^{2}}{2\sigma^{2}}} \right)-Normsinv \left( 1- \frac{p}{2} \right) \right)100;$$

Where Normsdist is the standard normal cumulative distribution, Normsinv is the inverse of the standard normal cumulative distribution, n is the sample size assuming equal sized groups, d is the difference between means, σ is the standard deviation and p is the significance level (p=0.00138 in our case after Bonferroni correction).

Since we lack equal sized groups (i.e. n1 > n2), we performed an estimation (n) using the following formula:

$$n=\frac{2kN}{\left( 1+k \right)^{2}}$$

Where k is the imbalance ratio (n_1_/n_2_) and N is the actual size (n_1_ + n_2_).

**Table S1. Sociodemographic, clinical, cognitive and neurophysiological values in chronic and minimally-treated patients.**

Data specified as %, simple distribution or pertaining to Mean (S.D.)

*p<0.01 (Mann-Whitney’s U test)

N/A = Not Applicable

|  | **Chronic patients (N=10)** | **Minimally-treated patients (N=21)** |
| --- | --- | --- |
| **Age *** | 44.60 (11.11) | 32.52 (10.67) |
| **Total IQ** | 83.25 (14.28) | 80.65 (13.85) |
| **PANSS positive** | 18.70 (5.19) | 21.11 (3.66) |
| **PANSS negative*** | 23.60 (4.72) | 17.50 (4.78) |
| **PANSS total** | 73.10 (12.91) | 76.78 (11.81) |
| **BACS verbal memory** | 38.78 (13.28) | 34.95 (12.10) |
| **BACS working memory** | 15.67 (7.21) | 16.53 (5.52) |
| **BACS motor speed** | 46.33 (9.87) | 51.30 (17.10) |
| **BACS verbal fluency** | 15.89 (4.29) | 16.45 (4.72) |
| **BACS processing speed** | 33.33 (15.99) | 37.95 (12.80) |
| **BACS problem solving** | 11.56 (6.86) | 12.83 (6.03) |
| **P3b valid segments (n)** | 48.20 (23.42) | 43.43 (20.13) |
| **Pz amplitude S2 (P3a; μV)** | 0.388 (0.925) | 1.015 (1.015) |
| **Pz amplitude S3 (P3b; μV)** | 0.099 (0.513) | 1.156 (1.811) |
| **Gamma Noise Power F3 (μV^2^)** | 0.013 (0.010) | 0.012 (0.013) |
| **Gamma Noise Power F4 (μV^2^)** | 0.015 (0.015) | 0.011 (0.013) |
| **Gamma Noise Power P3 (μV^2^)** | 0.013 (0.010) | 0.013 (0.010) |
| **Gamma Noise Power P4 (μV^2^)** | 0.015 (0.011) | 0.011 (0.005) |

**Table S2. Sociodemographic, clinical, cognitive and neurophysiological values according to NRG1 rs6994992 and rs3924999, and NRG3 rs10748842 genotypes considering only minimally-treated patients.**

No within-group significant differences were detected between patients or HC subgroups classified according to their genotype. Differences between minimally-treated patients and HC with the same SNP variation are displayed in the patient’s column. Data exhibited as Mean (S.D.)

* p<0.05; ** p<0.01; *** p<0.001; Significant results after Bonferroni correction (p<0.00138) are in bold-lettering (Mann-Whitney’s U test).

N/A = Not Applicable.

|  | **NRG1 rs6994992** | | | | **NRG1 rs3924999** | | | | **NRG3 rs10748842** | | | |
| --- | --- | --- | --- | --- | --- | --- | --- | --- | --- | --- | --- | --- |
|  | **Minimally-treated patients** | | **Healthy controls** | | **Minimally-treated patients** | | **Healthy controls** | | **Minimally-treated patients** | | **Healthy controls** | |
|  | **T+**  **(N=13)** | **T-**  **(N=8)** | **T+**  **(N=12)** | **T-**  **(N=11)** | **A+**  **(N=12)** | **A-**  **(N=9)** | **A+**  **(N=16)** | **A-**  **(N=7)** | **C+**  **(N=5)** | **C-**  **(N=16)** | **C+**  **(N=4)** | **C-**  **(N=19)** |
| **Age (years)** | 30.38  (9.42) | 36.00  (12.27) | 33.92  (14.86) | 29.73  (11.25) | 30.50  (11.14) | 35.22  (9.97) | 33.69  (13.42) | 27.86  (12.44) | 31.40  (3.91) | 32.88  (12.13) | 40.25  (21.22) | 30.16  (10.82) |
| **Total IQ** | 79.00  (16.78)** | 83.13  (8.17)** | 101.92  (11.91) | 106.27  (14.50) | **80.50**  **(14.53)***** | 80.88  (13.75)** | 104.19  (14.10) | 103.57  (11.47) | 81.50  (4.20)* | **80.44**  **(15.47)***** | 106.25  (14.34) | 103.53  (13.19) |
| **PANSS positive** | 20.83  (3.16) | 21.67  (4.80) | N/A | N/A | 21.40  (3.92) | 20.75  (3.54) | N/A | N/A | 19.25  (3.59) | 21.64  (3.63) | N/A | N/A |
| **PANSS negative** | 17.17  (4.41) | 18.17  (5.85) | N/A | N/A | 15.00  (3.33)* | 20.63  (4.60) | N/A | N/A | 16.75  (7.27) | 17.71  (4.18) | N/A | N/A |
| **PANSS total** | 75.83  (11.68) | 78.67  (12.93) | N/A | N/A | 71.10  (8.48)* | 83.88  (11.93) | N/A | N/A | 76.25  (14.61) | 76.93  (11.53) | N/A | N/A |
| **BACS verbal memory** | 32.45  (15.18)** | **38.38**  **(4.93)***** | 51.75  (8.71) | 58.27  (4.69) | **36.50**  **(12.93)***** | 32.29  (10.95)** | 55.50  (6.61) | 53.43  (10.18) | 37.50  (6.56) | **34.27**  **(13.30)***** | 50.25  (9.91) | 55.84  (7.07) |
| **BACS working memory** | 15.64  (6.64)* | 17.75  (3.54)* | 22.17  (4.34) | 22.18  (3.25) | **16.18**  **(5.02)***** | 17.00  (6.48) | 22.50  (3.18) | 21.43  (5.09) | 16.50  (3.70)* | 16.53  (6.02)** | 23.00  (1.41) | 22.00  (4.11) |
| **BACS motor speed** | 49.42  (19.48)* | 54.13  (13.51) | 65.33  (15.69) | 63.82  (14.60) | 54.50  (16.41) | 46.50  (18.07)** | 60.75  (14.23)* | 73.43  (13.10) | 56.75  (14.82) | 49.94  (17.79)** | 59.50  (23.74) | 65.68  (12.98) |
| **BACS verbal fluency** | **15.92**  **(5.65)***** | **17.25**  **(3.01)***** | 25.17  (5.37) | 25.82  (4.42) | **16.67**  **(5.43)***** | 16.13  (3.72)* | 27.13  (3.38)* | 21.71  (5.79) | 18.25  (1.89)* | **16.00**  **(5.14)***** | 27.75  (4.35) | 25.00  (4.91) |
| **BACS processing speed** | 38.36  (15.88)** | **37.38**  **(7.76)***** | 57.17  (11.47) | 60.09  (13.42) | **40.33**  **(15.02)***** | 33.86  (6.87)** | 60.13  (13.02) | 55.00  (10.21) | 38.50  (5.00)* | **37.80**  **(14.32)***** | 49.75  (5.44) | 60.42  (12.54) |
| **BACS problem solving** | 13.40  (5.72) | 12.13  (6.73)* | 16.92  (3.06) | 18.18  (3.16) | 12.55  (5.73)* | 13.29  (6.92) | 17.25  (3.32) | 18.14  (2.67) | 11.50  (5.26) | 13.21  (6.36)* | 15.75  (3.77) | 17.89  (2.92) |
| **P3b valid segments (n)** | 44.92  (18.23) | 41.00  (24.02)* | 53.25  (25.03) | 61.36  (24.33) | 43.75  (20.10) | 43.00  (21.38) | 54.94  (25.72) | 62.14  (22.41) | 52.00  (26.43) | 40.75  (17.94)* | 54.00  (17.42) | 57.79  (26.07) |
| **Pz amplitude S2 (P3a; μV)** | 1.280  (1.161) | 0.649  (1.298) | 1.485  (0.762) | 1.093  (1.179) | 1.076  (1.290) | 0.992  (1.204) | 1.210  (0.982) | 1.497  (1.025) | 0.883  (1.278) | 1.089  (1.245) | 1.114  (0.691) | 1.336  (1.043) |
| **Pz amplitude S3 (P3b; μV)** | 1.183  (1.696) | 1.175  (1.991) | 2.033  (0.680) | 1.827  (1.136) | 1.166  (1.574) | 1.197  (2.092) | 1.623  (0.873)** | 2.647  (0.532) | 0.754  (2.017) | 1.313  (1.729) | 1.915  (0.400) | 1.939  (0.993) |
| **Gamma Noise Power F3 (μV^2^)** | 0.012  (0.014) | 0.011  (0.011) | 0.007  (0.003) | 0.013  (0.022) | 0.013  (0.016) | 0.009  (0.006) | 0.007  (0.005) | 0.016  (0.026) | 0.014  (0.014) | 0.011  (0.013) | 0.004  (0.000) | 0.011  (0.016) |
| **Gamma Noise Power F4 (μV^2^)** | 0.008  (0.005) | 0.015  (0.020) | 0.006  (0.003) | 0.011  (0.013) | 0.013  (0.016) | 0.007  (0.003) | 0.007  (0.004) | 0.012  (0.016) | 0.008  (0.005) | 0.012  (0.014) | 0.005  (0.001) | 0.009  (0.010) |
| **Gamma Noise Power P3 (μV^2^)** | 0.012  (0.010)* | 0.015  (0.011)* | 0.006  (0.003) | 0.007  (0.004) | 0.012  (0.011)* | 0.013  (0.010)* | 0.006  (0.004) | 0.006  (0.002) | 0.014  (0.009)* | 0.012  (0.011)* | 0.004  (0.002) | 0.007  (0.004) |
| **Gamma Noise Power P4 (μV^2^)** | 0.011  (0.006)* | 0.010  (0.003) | 0.007  (0.005) | 0.007  (0.004) | 0.012  (0.006)* | 0.010  (0.004) | 0.007  (0.004) | 0.009  (0.006) | 0.009  (0.005) | 0.011  (0.005)* | 0.005  (0.004) | 0.008  (0.005) |

**Table S3. Power values for the different comparison analyses at p<0.00138 (Bonferroni corrected) significance level.**

* p<0.05; ** p<0.01; *** p<0.001; Significant results after Bonferroni correction (p<0.00138) are in bold-lettering (Mann-Whitney’s U test).

P: Patients; HC: Healthy Controls.

|  | **NRG1 rs6994992** | | | | **NRG1 rs3924999** | | | | **NRG3 rs10748842** | | | |
| --- | --- | --- | --- | --- | --- | --- | --- | --- | --- | --- | --- | --- |
|  | **T+ vs T-** | | **P vs HC** | | **A+ vs A-** | | **P vs HC** | | **C+ vs C-** | | **P vs HC** | |
|  | **P** | **HC** | **T+** | **T-** | **P** | **HC** | **A+** | **A-** | **P** | **HC** | **C+** | **C-** |
| **Sample size for unequal sized groups (n1:n2)** | 21:10 | 12:11 | 21:12 | 10:11 | 18:13 | 16:7 | 18:16 | 13:7 | 8:23 | 4:19 | 8:4 | 23:19 |
| **Imbalance ratio** | 2.10 | 1.09 | 1.75 | 1.10 | 1.38 | 2.29 | 1.13 | 1.86 | 2.88 | 4.75 | 2.00 | 1.21 |
| **Sample size assuming equal sized groups (per group)** | 13 | 11 | 15 | 10 | 15 | 9 | 16 | 9 | 11 | 6 | 5 | 20 |
| **BACS verbal memory** | 0.30 | 17.97 | 76.14** | **99.99***** | 0.76 | 0.37 | **97.17***** | 74.50* | 0.20 | 1.98 | 12.17 | **99.91***** |
| **BACS working memory** | 1.31 | 0.07 | 47.99** | 31.06* | 0.15 | 0.40 | 67.29** | 10.20 | 0.08 | 0.51 | 53.20* | 55.93** |
| **BACS motor speed** | 0.85 | 0.15 | 34.34* | 7.29 | 0.85 | 10.93 | 6.44 | 67.80** | 0.08 | 0.45 | 0.75 | 55.75** |
| **BACS verbal fluency** | 0.12 | 0.19 | **90.50***** | **97.66***** | 0.27 | 24.47 | **100.00***** | 12.41* | 0.66 | 1.50 | 81.76** | **99.25***** |
| **BACS processing speed** | 0.42 | 0.40 | 64.31** | **96.97***** | 0.30 | 1.19 | **83.20***** | 70.08** | 0.10 | 12.65 | 9.52 | **98.29***** |
| **BACS problem solving** | 2.22 | 1.23 | 17.17 | 57.21* | 1.12 | 0.51 | 58.62** | 16.46 | 0.17 | 1.83 | 1.31 | 75.43** |
| **Pz amplitude S2 (P3a)** | 0.58 | 1.22 | 4.46 | 0.86 | 0.21 | 0.48 | 0.68 | 3.90 | 0.31 | 0.29 | 0.69 | 2.13 |
| **Pz amplitude S3 (P3b)** | 0.24 | 0.38 | 51.95** | 2.75 | 0.13 | 45.75 | 13.17* | 49.63* | 0.24 | 0.08 | 13.42* | 25.84* |
| **Gamma Noise Power F3** | 0.15 | 1.91 | 8.49 | 0.17 | 3.94 | 2.46 | 13.25* | 1.25 | 0.25 | 4.62 | 20.41* | 0.07 |
| **Gamma Noise Power F4** | 6.69 | 4.15 | 8.49 | 1.14 | 15.67 | 1.63 | 14.83 | 1.87 | 0.21 | 2.62 | 4.36 | 0.43 |
| **Gamma Noise Power P3** | 1.68 | 0.57 | 17.97* | 26.00** | 0.19 | 0.07 | 35.56** | 25.68 | 0.16 | 7.12 | 27.04* | 17.36** |
| **Gamma Noise Power P4** | 0.25 | 0.07 | 13.74* | 11.29 | 8.49 | 0.94 | 27.83** | 0.07 | 0.21 | 2.05 | 9.66 | 10.51* |
